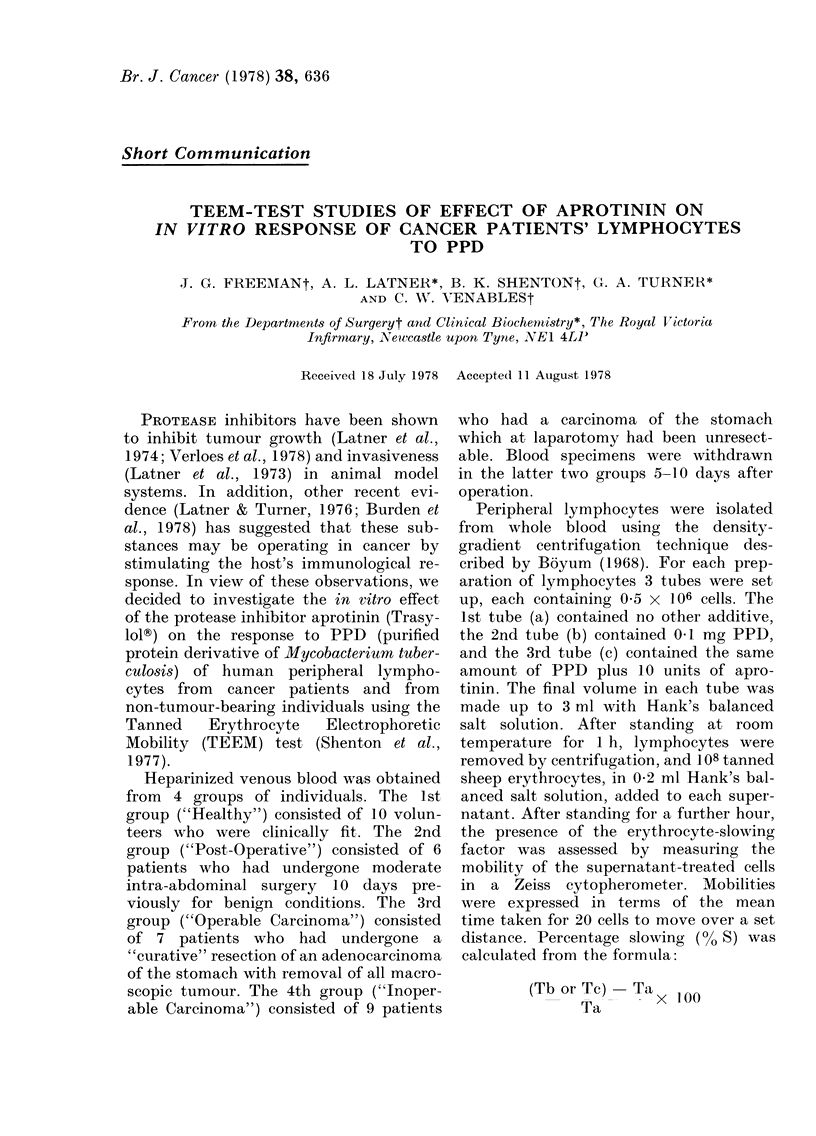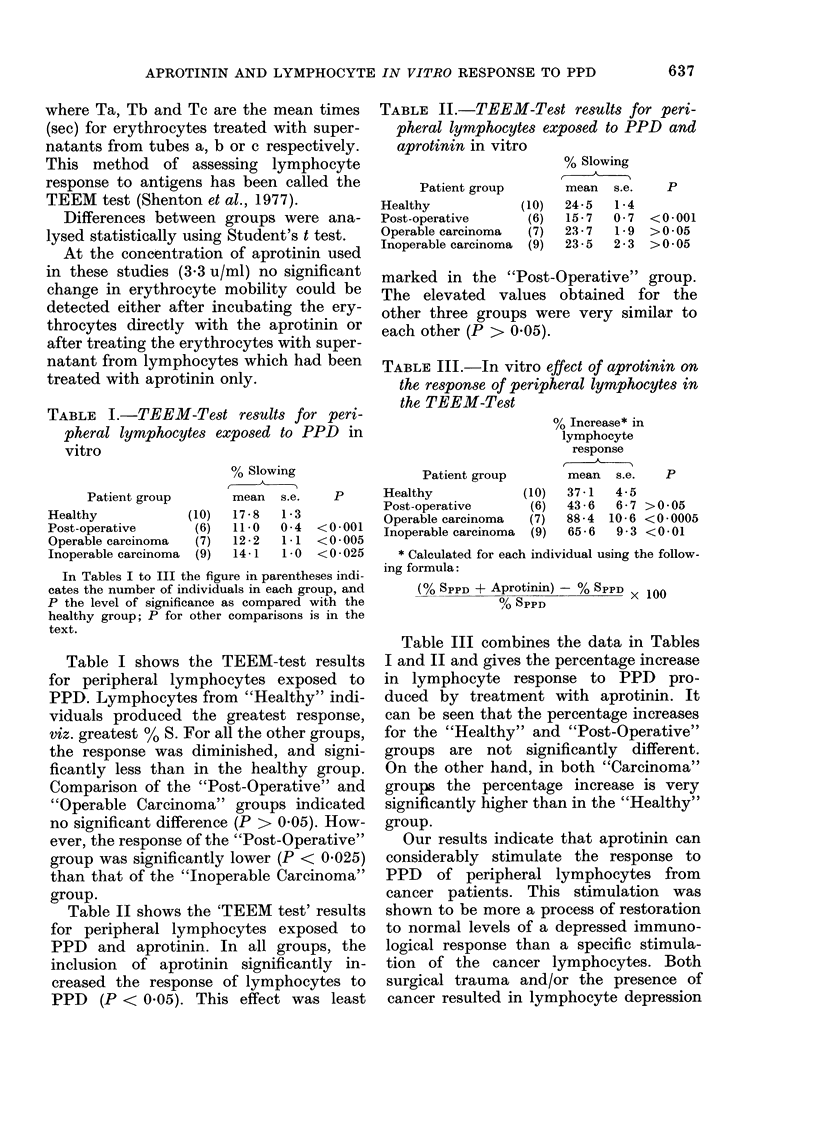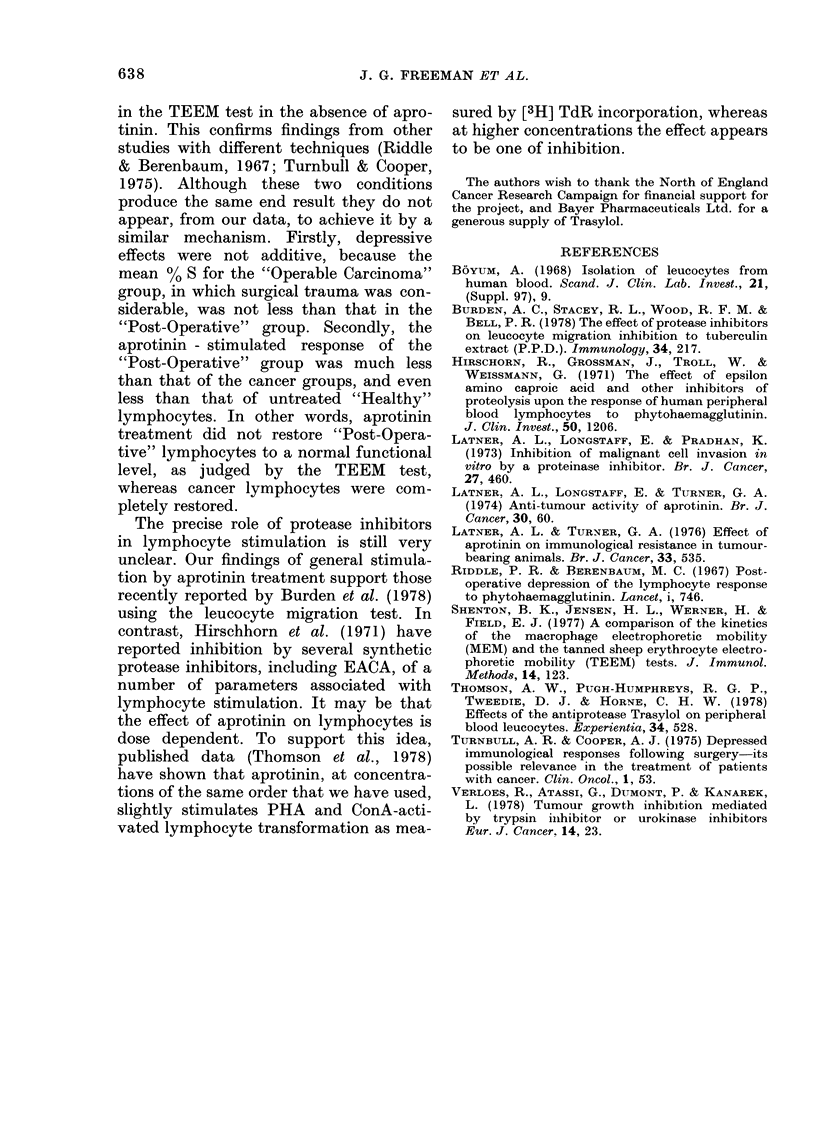# TEEM-test studies on effect of aprotinin on in vitro response of cancer patients' lymphocytes to PPD.

**DOI:** 10.1038/bjc.1978.262

**Published:** 1978-11

**Authors:** J. G. Freeman, A. L. Latner, B. K. Shenton, G. A. Turner, C. W. Venables


*Br. J. Cancer* (1978) **38**, 636

***Short Communication***

### TEEM-TEST STUDIES OF EFFECT OF APROTININ ON *IN VITRO* RESPONSE OF CANCER PATIENTS' LYMPHOCYTES TO PPD

J. G. FREEMAN†, A. L. LATNER*, B. K. SHENTON†, G. A. TURNER* AND C. W. VENABLES†

*From the Departments of Surgery† and Clinical Biochemistry*, The Royal Victoria Infirmary, Newcastle upon Tyne, NE1 4LP*

Received 18 July 1978 Accepted 11 August 1978

PROTEASE inhibitors have been shown to inhibit tumour growth (Latner *et al.*, 1974; Verloes *et al.*, 1978) and invasiveness (Latner *et al.*, 1973) in animal model systems. In addition, other recent evidence (Latner & Turner, 1976; Burden *et al.*, 1978) has suggested that these substances may be operating in cancer by stimulating the host's immunological response. In view of these observations, we decided to investigate the *in vitro* effect of the protease inhibitor aprotinin (Trasylol®) on the response to PPD (purified protein derivative of *Mycobacterium tuberculosis*) of human peripheral lymphocytes from cancer patients and from non-tumour-bearing individuals using the Tanned Erythrocyte Electrophoretic Mobility (TEEM) test (Shenton *et al.*, 1977).

Heparinized venous blood was obtained from 4 groups of individuals. The 1st group ("Healthy") consisted of 10 volunteers who were clinically fit. The 2nd group ("Post-Operative") consisted of 6 patients who had undergone moderate intra-abdominal surgery 10 days previously for benign conditions. The 3rd group ("Operable Carcinoma") consisted of 7 patients who had undergone a "curative" resection of an adenocarcinoma of the stomach with removal of all macroscopic tumour. The 4th group ("Inoperable Carcinoma") consisted of 9 patients who had a carcinoma of the stomach which at laparotomy had been unresectable. Blood specimens were withdrawn in the latter two groups 5–10 days after operation.

Peripheral lymphocytes were isolated from whole blood using the density-gradient centrifugation technique described by Böyum (1968). For each preparation of lymphocytes 3 tubes were set up, each containing $0.5 \times 10^6$ cells. The 1st tube (a) contained no other additive, the 2nd tube (b) contained 0·1 mg PPD, and the 3rd tube (c) contained the same amount of PPD plus 10 units of aprotinin. The final volume in each tube was made up to 3 ml with Hank's balanced salt solution. After standing at room temperature for 1 h, lymphocytes were removed by centrifugation, and $10^8$ tanned sheep erythrocytes, in 0·2 ml Hank's balanced salt solution, added to each supernatant. After standing for a further hour, the presence of the erythrocyte-slowing factor was assessed by measuring the mobility of the supernatant-treated cells in a Zeiss cytopherometer. Mobilities were expressed in terms of the mean time taken for 20 cells to move over a set distance. Percentage slowing (% S) was calculated from the formula:

$$\frac{(\mathrm{Tb\ or\ Tc}) - \mathrm{Ta}}{\mathrm{Ta}} \times 100$$

where Ta, Tb and Tc are the mean times (sec) for erythrocytes treated with supernatants from tubes a, b or c respectively. This method of assessing lymphocyte response to antigens has been called the TEEM test (Shenton *et al.*, 1977).

Differences between groups were analysed statistically using Student's *t* test.

At the concentration of aprotinin used in these studies (3·3 u/ml) no significant change in erythrocyte mobility could be detected either after incubating the erythrocytes directly with the aprotinin or after treating the erythrocytes with supernatant from lymphocytes which had been treated with aprotinin only.

TABLE I.—*TEEM-Test results for peripheral lymphocytes exposed to PPD* in vitro

| Patient group | | % Slowing mean | s.e. | *P* |
|---|---|---|---|---|
| Healthy | (10) | 17·8 | 1·3 | |
| Post-operative | (6) | 11·0 | 0·4 | <0·001 |
| Operable carcinoma | (7) | 12·2 | 1·1 | <0·005 |
| Inoperable carcinoma | (9) | 14·1 | 1·0 | <0·025 |

In Tables I to III the figure in parentheses indicates the number of individuals in each group, and *P* the level of significance as compared with the healthy group; *P* for other comparisons is in the text.

Table I shows the TEEM-test results for peripheral lymphocytes exposed to PPD. Lymphocytes from "Healthy" individuals produced the greatest response, *viz.* greatest % S. For all the other groups, the response was diminished, and significantly less than in the healthy group. Comparison of the "Post-Operative" and "Operable Carcinoma" groups indicated no significant difference ($P > 0{\cdot}05$). However, the response of the "Post-Operative" group was significantly lower ($P < 0{\cdot}025$) than that of the "Inoperable Carcinoma" group.

Table II shows the 'TEEM test' results for peripheral lymphocytes exposed to PPD and aprotinin. In all groups, the inclusion of aprotinin significantly increased the response of lymphocytes to PPD ($P < 0{\cdot}05$). This effect was least marked in the "Post-Operative" group. The elevated values obtained for the other three groups were very similar to each other ($P > 0{\cdot}05$).

TABLE II.—*TEEM-Test results for peripheral lymphocytes exposed to PPD and aprotinin* in vitro

| Patient group | | % Slowing mean | s.e. | *P* |
|---|---|---|---|---|
| Healthy | (10) | 24·5 | 1·4 | |
| Post-operative | (6) | 15·7 | 0·7 | <0·001 |
| Operable carcinoma | (7) | 23·7 | 1·9 | >0·05 |
| Inoperable carcinoma | (9) | 23·5 | 2·3 | >0·05 |

TABLE III.—In vitro *effect of aprotinin on the response of peripheral lymphocytes in the TEEM-Test*

| Patient group | | % Increase* in lymphocyte response mean | s.e. | *P* |
|---|---|---|---|---|
| Healthy | (10) | 37·1 | 4·5 | |
| Post-operative | (6) | 43·6 | 6·7 | >0·05 |
| Operable carcinoma | (7) | 88·4 | 10·6 | <0·0005 |
| Inoperable carcinoma | (9) | 65·6 | 9·3 | <0·01 |

* Calculated for each individual using the following formula:

$$\frac{(\%\ S_{PPD} + \text{Aprotinin}) - \%\ S_{PPD}}{\%\ S_{PPD}} \times 100$$

Table III combines the data in Tables I and II and gives the percentage increase in lymphocyte response to PPD produced by treatment with aprotinin. It can be seen that the percentage increases for the "Healthy" and "Post-Operative" groups are not significantly different. On the other hand, in both "Carcinoma" groups the percentage increase is very significantly higher than in the "Healthy" group.

Our results indicate that aprotinin can considerably stimulate the response to PPD of peripheral lymphocytes from cancer patients. This stimulation was shown to be more a process of restoration to normal levels of a depressed immunological response than a specific stimulation of the cancer lymphocytes. Both surgical trauma and/or the presence of cancer resulted in lymphocyte depression in the TEEM test in the absence of aprotinin. This confirms findings from other studies with different techniques (Riddle & Berenbaum, 1967; Turnbull & Cooper, 1975). Although these two conditions produce the same end result they do not appear, from our data, to achieve it by a similar mechanism. Firstly, depressive effects were not additive, because the mean % S for the "Operable Carcinoma" group, in which surgical trauma was considerable, was not less than that in the "Post-Operative" group. Secondly, the aprotinin - stimulated response of the "Post-Operative" group was much less than that of the cancer groups, and even less than that of untreated "Healthy" lymphocytes. In other words, aprotinin treatment did not restore "Post-Operative" lymphocytes to a normal functional level, as judged by the TEEM test, whereas cancer lymphocytes were completely restored.

The precise role of protease inhibitors in lymphocyte stimulation is still very unclear. Our findings of general stimulation by aprotinin treatment support those recently reported by Burden *et al.* (1978) using the leucocyte migration test. In contrast, Hirschhorn *et al.* (1971) have reported inhibition by several synthetic protease inhibitors, including EACA, of a number of parameters associated with lymphocyte stimulation. It may be that the effect of aprotinin on lymphocytes is dose dependent. To support this idea, published data (Thomson *et al.*, 1978) have shown that aprotinin, at concentrations of the same order that we have used, slightly stimulates PHA and ConA-activated lymphocyte transformation as measured by [$^3$H] TdR incorporation, whereas at higher concentrations the effect appears to be one of inhibition.

The authors wish to thank the North of England Cancer Research Campaign for financial support for the project, and Bayer Pharmaceuticals Ltd. for a generous supply of Trasylol.

### REFERENCES

BÖYUM, A. (1968) Isolation of leucocytes from human blood. *Scand. J. Clin. Lab. Invest.*, **21**, (Suppl. 97), 9.

BURDEN, A. C., STACEY, R. L., WOOD, R. F. M. & BELL, P. R. (1978) The effect of protease inhibitors on leucocyte migration inhibition to tuberculin extract (P.P.D.). *Immunology*, **34**, 217.

HIRSCHORN, R., GROSSMAN, J., TROLL, W. & WEISSMANN, G. (1971) The effect of epsilon amino caproic acid and other inhibitors of proteolysis upon the response of human peripheral blood lymphocytes to phytohaemagglutinin. *J. Clin. Invest.*, **50**, 1206.

LATNER, A. L., LONGSTAFF, E. & PRADHAN, K. (1973) Inhibition of malignant cell invasion *in vitro* by a proteinase inhibitor. *Br. J. Cancer*, **27**, 460.

LATNER, A. L., LONGSTAFF, E. & TURNER, G. A. (1974) Anti-tumour activity of aprotinin. *Br. J. Cancer*, **30**, 60.

LATNER, A. L. & TURNER, G. A. (1976) Effect of aprotinin on immunological resistance in tumour-bearing animals. *Br. J. Cancer*, **33**, 535.

RIDDLE, P. R. & BERENBAUM, M. C. (1967) Post-operative depression of the lymphocyte response to phytohaemagglutinin. *Lancet*, i, 746.

SHENTON, B. K., JENSEN, H. L., WERNER, H. & FIELD, E. J. (1977) A comparison of the kinetics of the macrophage electrophoretic mobility (MEM) and the tanned sheep erythrocyte electrophoretic mobility (TEEM) tests. *J. Immunol. Methods*, **14**, 123.

THOMSON, A. W., PUGH-HUMPHREYS, R. G. P., TWEEDIE, D. J. & HORNE, C. H. W. (1978) Effects of the antiprotease Trasylol on peripheral blood leucocytes. *Experientia*, **34**, 528.

TURNBULL, A. R. & COOPER, A. J. (1975) Depressed immunological responses following surgery—its possible relevance in the treatment of patients with cancer. *Clin. Oncol.*, **1**, 53.

VERLOES, R., ATASSI, G., DUMONT, P. & KANAREK, L. (1978) Tumour growth inhibition mediated by trypsin inhibitor or urokinase inhibitors *Eur. J. Cancer*, **14**, 23.